# School-based mindfulness education and children’s emotion regulation: the mediating role of executive function

**DOI:** 10.3389/fpsyg.2026.1760807

**Published:** 2026-03-04

**Authors:** Xuhong Wang, Xuefei Dong

**Affiliations:** 1 Future Industries College, Xi'an Peihua University, Xi’an, Shaanxi, China; 2 International Education College, Xi'an Peihua University, Xi’an, Shaanxi, China

**Keywords:** child emotion regulation, executive function, mediation mechanism, mindfulness education, primary school children

## Abstract

**Background:**

School-based mindfulness education has been increasingly implemented to support children’s socio-emotional development; however, the cognitive mechanisms underlying its effects on emotion regulation remain insufficiently clarified. This study examined whether mindfulness-based education improves children’s emotion regulation and whether executive function mediates this association.

**Methods:**

In a randomized controlled trial, 150 children aged 8–10 years were assigned to either a mindfulness education group or a waitlist control group. The 8-week program assessed emotion regulation (ERC, Emotion Management Scale, Disappointing Gift Task) and executive function (Hearts and Flowers, Digit Span, WCST).

**Results:**

The mindfulness group showed significantly greater improvements in emotion regulation strategies (*F* = 15.37, *p* < 0.001) and all executive function components. Mediation analysis revealed significant indirect effects through inhibitory control, working memory, and cognitive flexibility, accounting for 53.8% of the total effect.

**Conclusion:**

Mindfulness education enhances children’s emotion regulation, both directly and through improvements in executive function, supporting its implementation in school mental health programs.

## 1 Introduction

In recent years, studies on mindfulness interventions in children’s emotion regulation have made significant progress. School-based mindfulness-based interventions (MBSIs) have been associated with small-to-moderate improvements in children’s socio-emotional outcomes, including aspects of emotion regulation, although effects vary by program format and study design (Zenner et al., 2014; Phan et al., 2022). Scholars explored the process by which mindful parenting and mindfulness-based training influenced children’s emotion regulation, thereby providing important evidence for the developmental role of mindfulness in childhood.

Chan and Tsui (2025) studied the impact of mindful parenting on children with autism spectrum disorder’s emotion regulation. The longitudinal results showed that mindful parenting had positive effects on children with special needs, improving their emotion regulation and behavioral adaptation. Kholghi et al. (2025) studied the impact of mindfulness-based cognitive therapy on children’s anxiety and emotion regulation based on a randomized controlled design. The results showed that the improvement in attention control mediated the gain in emotion regulation. Tao et al. (2025) studied the association between mindful parenting and children’s internalizing problems. The questionnaire data showed that children’s emotion regulation mediated the association with mindful parenting. Parental gender moderated the mediation process, thereby enriching the understanding of mindfulness from the perspective of family systems. Chhangur et al. (2025) investigated how negative affect influences the effectiveness of mindful parenting, as measured with EEG indices and ecological momentary assessment. EEG frontal alpha asymmetry moderated the relationship between parenting behavior and children’s externalizing problems. Lin et al. (2025) designed an emotional mindfulness parenting intervention and then evaluated its effects in a randomized controlled trial. Results indicated that an emotional mindfulness parenting intervention improved preschoolers’ emotion regulation, and that parental stress mediated the intervention’s effect on preschoolers.

Wang et al. (2025) investigated mindfulness in families of children with autism. A daily diary method was used, and results indicated that everyday parenting experiences influenced marital relations through emotional experiences, and that trait mindfulness moderated the relationship. Gong et al. (2025) conducted a randomized controlled trial over 6 weeks with migrant children. Results indicated that mindfulness training improved preschoolers’ emotional regulation, decreased mind wandering, and promoted inner calm. Julian et al. (2025) focused on the relationship between childhood maltreatment and well-being, revealing through cross-sectional data that self-compassion and mindfulness sequentially mediated emotion regulation, providing insights for trauma-focused interventions. He et al. (2025) investigated maternal mindful parenting and toddlers’ emotion regulation using multilevel modeling, showing that depressive symptoms mediated the effect while the parent–child relationship moderated the pathway. Elzohairy et al. (2024) examined the efficacy of mindfulness training for children with ADHD and found improvements in attention and impulse control, with family involvement moderating the outcomes. Lan et al. (2024) explored the combined effects of mindfulness and life-skills training on migrant children, revealing that the integrated intervention significantly enhanced emotion regulation and reduced anxiety symptoms. Sansone (2024) proposed a theoretical framework of mindful parenting, highlighting its pivotal role in children’s emotional regulation and human flourishing, and providing conceptual guidance for future inquiry.

Despite substantial empirical evidence linking mindfulness interventions with improved emotional regulation in children, the specific pathways through which executive function operates in this relationship remain insufficiently examined. Particularly among typically developing school-aged children, few studies have systematically tested the mediating roles of the three core executive components—inhibitory control, working memory, and cognitive flexibility—within a unified model. Addressing this theoretical gap, the present randomized controlled study systematically investigates the promotive effects of mindfulness-based education on children’s emotion regulation, with a particular focus on elucidating the multiple mediation mechanisms of executive function. This research aims to provide novel empirical evidence to refine theoretical models of children’s emotional competence development.

## 2 Materials and methods

### 2.1 Participants and study design

#### 2.1.1 Recruitment and eligibility criteria

A total of 150 children aged 8–10 years were recruited from two public primary schools in Xi’an, Shaanxi Province, China. The study targeted typically developing children from a community sample to examine the general promotive influence of school-based mindfulness education on emotion regulation.

Inclusion criteria were as follows: (1) aged 8–10 years; (2) both the child and his/her parent or legal guardian were willing to participate; and (3) typical intellectual and language development sufficient to perform the experimental tasks. Exclusion criteria were as follows: (1) a clinically diagnosed severe neurodevelopmental disorder (e.g., autism spectrum disorder), psychiatric disorder, or severe physical illness; and (2) prior participation in mindfulness-based or cognitive-behavioral interventions lasting more than 6 consecutive months.

#### 2.1.2 Randomization and blinding procedures

The study used a two-arm, parallel-group, randomized controlled design with blinded outcome assessment. Individual-level randomization was employed, with eligible participants assigned to the mindfulness education group or the waitlist control group (1:1), regardless of their classroom or school affiliation. The allocation sequence was generated by an independent researcher using a computer-based random number generator; this researcher was not involved in participant recruitment, intervention delivery, outcome assessment, or data analysis. Sequence envelopes were concealed, sequentially numbered, and opaque to achieve allocation concealment.

Due to the nature of the intervention, participants and instructors could not be blinded. However, outcome assessors responsible for posttest data collection and independent coders analyzing behavioral video recordings were blinded to group allocation, thereby minimizing measurement bias to the greatest extent possible.

### 2.2 Sample size estimation

*A priori* power analysis was conducted using G*Power 3.1 software. With the Emotion Regulation Checklist (ERC) score as the primary outcome, a medium effect size (*d* = 0.6), alpha level of 0.05, and statistical power of 0.90, the analysis indicated a required sample size of 60 participants per group. To account for an estimated 20% attrition rate, the final target sample size was set at 150 participants. Executive function capacities are closely linked to children’s emotion regulation and emotion dysregulation across development (Groves et al., 2021; Pozo-Rodríguez et al., 2026).

### 2.3 Intervention protocol

#### 2.3.1 Mindfulness education group: intervention content and procedure

Children in the mindfulness education group received a structured eight-week mindfulness curriculum (one 45-min session per week) adapted from established child-focused mindfulness interventions. Each session followed a standardized structure: 5-min check-in and settling, 10-min breathing-awareness practice, 10-min body scan, 15-min emotion-awareness and labeling exercises, and 5-min closing reflection. Certified instructors with formal training in mindfulness-based interventions (MBSR/MBCT) delivered the program. Home practice assignments of 5–10 min daily were encouraged to reinforce skills.

#### 2.3.2 Control group activities

Children in the waitlist control group were assigned to the waitlist condition and also concurrently participated in non-mindfulness group activities of similar duration and frequency (8 weeks, once a week, 45 min. Per session; e.g., drawing, handicrafts, and sharing stories), providing valuable social and recreational opportunities. All activities were designed so they did not contain any mindfulness-, cognitive-strategy-, or emotion-management-related components assigned as null, thereby allowing any between-group variance to be attributed to the intervention itself rather than to nonspecific group interaction or teacher exposure effects.

#### 2.3.3 Intervention Fidelity and adherence monitoring

To ensure high fidelity and consistency of implementation, we implemented strict quality control procedures. All instructors completed the same training and successfully passed competency demonstrations prior to instructing. We randomly video-reviewed 20% of sessions and used a Course Fidelity Checklist developed by the research team. Instructors had to achieve an average fidelity score of 90% or higher on this checklist to meet fidelity requirements.

Participant adherence was monitored through session attendance records and homework completion logs. Data from participants who attended fewer than 80% of the total sessions were excluded from the final analyses to ensure the reliability and validity of the intervention effects.

#### 2.3.4 Study measures

#### 2.3.5 Emotion regulation assessment

(1) Emotion regulation strategies: The parent version of the *Emotion Regulation Checklist (ERC)* was used to evaluate children’s emotion regulation strategies. The scale comprises 24 items rated on a 5-point Likert scale (1 = never, 5 = always), with higher scores indicating more adaptive use of emotion regulation strategies. In the present study, the Cronbach’s *α* for this scale was 0.86.
(2) Externalizing emotional behaviors: The teacher version of the *Children’s Emotion Management Scale* was administered to assess emotional externalization. This scale contains 30 items rated on a 4-point scale (1 = not at all, 4 = very much), with total scores ranging from 30 to 120; higher scores indicate more pronounced externalizing problems. The internal consistency reliability in this study was 0.91.
(3) Emotional behavioral responses: The *Disappointing Gift Paradigm* was employed to elicit mild disappointment in a standardized procedure, and children’s behavioral responses were video-recorded. Two independent raters, blinded to group allocation, coded the videos using a pre-specified manual. Inter-rater reliability (ICC) was 0.87. This task primarily assessed two dimensions: children’s emotion-masking ability and the intensity of negative emotional expression.

#### 2.3.6 Executive function assessment

(1) Inhibitory control and cognitive flexibility: The *Hearts and Flowers* task was used, comprising congruent, incongruent, and mixed conditions. Performance was evaluated using reaction time and accuracy. The mean reaction time difference in incongruent and mixed conditions served as a key indicator of inhibitory control, while accuracy reflected overall task efficiency.
(2) Working memory: The *Digit Span Task* required children to recall sequences of increasing length both forwards and backwards. Forward spans ranged from 2 to 9 digits, and backward spans from 2 to 8 digits. The most extended correctly recalled sequence determined the score for each condition, and the sum of forward and backward spans determined the total working memory score.
(3) Cognitive flexibility (set-shifting): The *Children’s Wisconsin Card Sorting Test (WCST)*, comprising 64 stimulus cards, was used to assess set-shifting ability through rule-change feedback. Primary metrics included the number of completed categories, perseverative errors, and the conceptual-level percentage, with perseverative errors serving as the core index of cognitive flexibility.

#### 2.3.7 Covariate data collection

(1) Demographic information: Basic demographic data—including age, gender, and parental highest education level (categorized as junior high school or below, senior high school, university, and graduate level or above)—were collected via a self-designed questionnaire. These variables were included as covariates in subsequent analyses to control for potential confounding effects.
(2) Family environment assessment: The *Family Assessment Device (FAD)* was used to evaluate family functioning. The scale contains 60 items rated on a 4-point scale (1 = strongly disagree, 4 = strongly agree) covering seven dimensions, including problem-solving, communication, and role allocation. Higher total scores indicate healthier family functioning. Cronbach’s *α* in this study was 0.89. Family functioning was assessed because family emotional climate and communication are associated with children’s self-regulatory capacities (including emotion regulation and executive functioning) and may confound intervention effects in school-based trials (Qiao et al., 2024; Cullen et al., 2025; Ryan et al., 2025).

### 2.4 Statistical analysis

#### 2.4.1 Primary and secondary outcomes

The total score on the parent-reported ERC was selected as the outcome measure to reflect the primary effect of the intervention. In addition, we defined the teacher-reported emotional management scores and performance on executive function tasks as secondary measures to assess intervention effects.

#### 2.4.2 Intervention effect analysis

To evaluate intervention effects, analyses of covariance (ANCOVA) were conducted to compare post-intervention outcomes between groups. In each model, posttest scores served as the dependent variable, group allocation (mindfulness education vs. control) as the independent variable, and baseline scores of the corresponding measures, as well as covariates showing baseline imbalance (e.g., parental education level), were included as control variables. This approach enhanced the statistical power and precision of the effect estimates.

#### 2.4.3 Mediation analysis

To examine whether executive function mediated the effect of the mindfulness intervention on changes in emotion regulation, a multiple mediation model was tested. Group allocation (mindfulness education vs. control) was entered as the independent variable, post-intervention ERC total score (controlling for baseline ERC) as the dependent variable, and post-intervention inhibitory control (Hearts and Flowers accuracy), working memory (Digit Span total score), and cognitive flexibility (WCST perseverative errors, reverse coded) as parallel mediators. Age, gender, parental education, family functioning, and baseline scores of all mediators and outcome variables were included as covariates. Bias-corrected bootstrapping with 5,000 resamples was used to estimate indirect effects and their 95% confidence intervals. Mediation analyses were conducted using PROCESS macro (Model 4) in SPSS, with bias-corrected bootstrapping (5,000 resamples).

### 2.5 Ethical considerations

This study was conducted in accordance with the ethical standards of the institutional and national research committees and with the 1964 Helsinki Declaration and its later amendments. Ethical review and approval was not required for this study on human participants in accordance with the local legislation and institutional requirements. The study protocol was reviewed by the Ethics Committee of the Future Industries College, Xi’an Peihua University, Xi’an, China, which confirmed that the project constituted minimal-risk research embedded in regular classroom activities and therefore met the criteria for exemption from formal ethics review. Written informed consent was obtained from the legal guardians of all participating children, and children provided assent prior to participation.

## 3 Results

### 3.1 Participant flow and baseline characteristics

**Figure 1:**
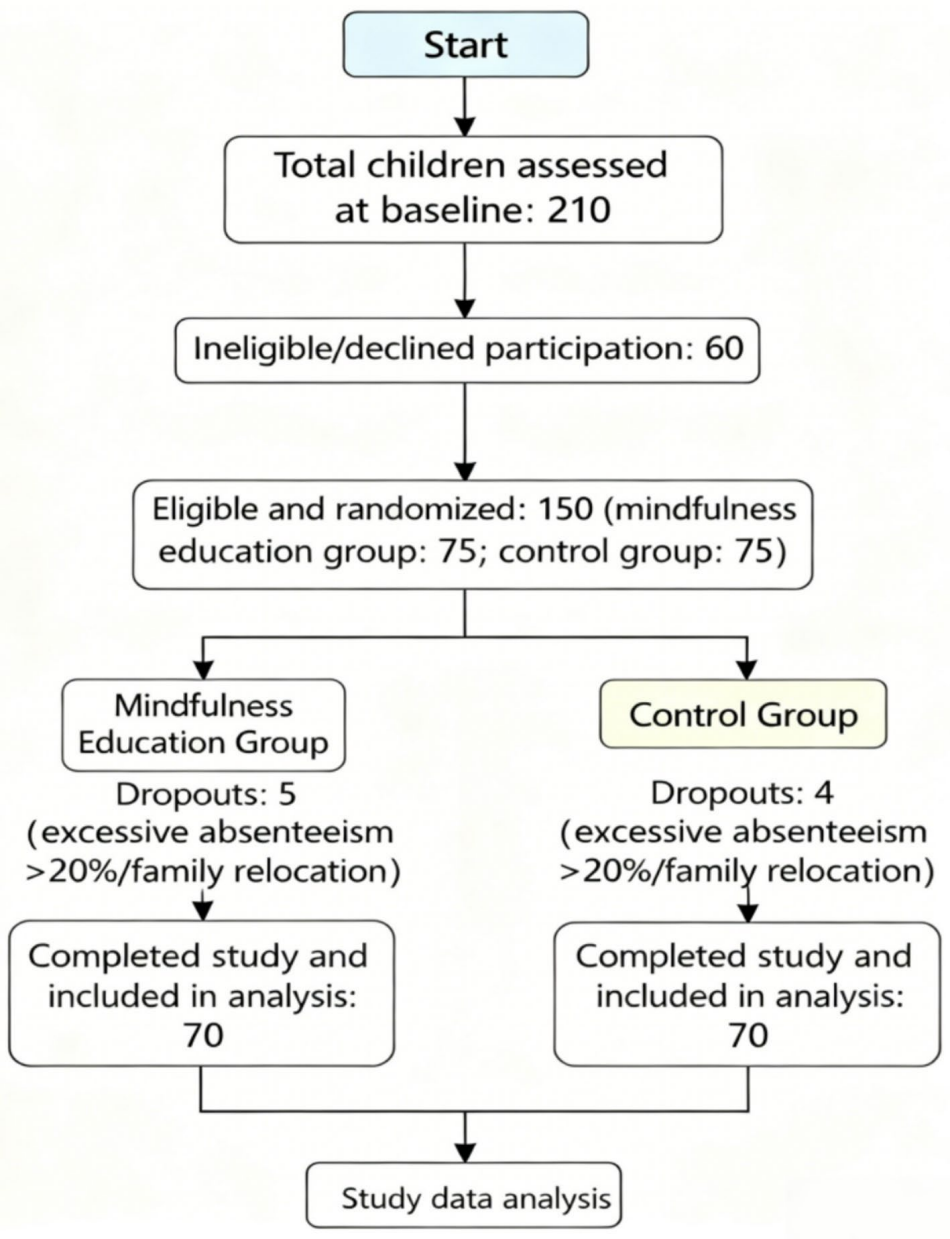
Participant flow through the randomized controlled trial.

As shown in the participant flow diagram (Figure 1), a total of 210 children were assessed for eligibility at baseline. A total of 150 eligible children were randomly assigned to the mindfulness education group (*n* = 75) or control group (n = 75). Five children in the mindfulness group and 4 children in the control group dropped out of the intervention due to excessive absenteeism (>20% sessions) or family relocation. In total, 140 children in both groups completed the full study protocol and were included in the analyses (70 children in each group).

As shown in Table 1, at baseline, there were no statistically significant differences between the two groups in terms of demographic variables (age, gender, parental education level), the primary outcome measure (score of emotion regulation strategy), the two dimensions of the secondary outcome measure (emotion management), or family environment covariates (all *p* > 0.05). This suggests that the randomization procedure was properly carried out and that the two groups were balanced at baseline, providing a valid basis for subsequent evaluation.

**Table 1:** Baseline characteristics of participants by group.

Characteristic	Mindfulness group (*n* = 70)	Control group (*n* = 70)	*p*-value
Demographics			
Age (years), mean (SD)	9.1 (0.8)	9.0 (0.7)	0.45
Gender (female), n (%)	35 (50.0)	34 (48.6)	0.86
Parental education (college or above), *n* (%)	42 (60.0)	45 (64.3)	0.59
Primary outcome			
ERC score, mean (SD)	78.5 (12.3)	76.8 (11.9)	0.38
Secondary outcomes			
Emotion regulation			
Emotion Management Scale (teacher), mean (SD)	65.2 (15.1)	67.1 (14.5)	0.44
Disappointing Gift Task (negative expression score), mean (SD)	3.8 (1.2)	3.9 (1.1)	0.58
Executive function			
Hearts and Flowers (mixed condition accuracy %), mean (SD)	75.4 (10.2)	74.1 (9.8)	0.42
Digit Span Task (total score), mean (SD)	12.5 (3.1)	12.8 (2.9)	0.53
WCST (perseverative errors), mean (SD)	15.2 (4.5)	14.8 (4.9)	0.59
Covariate			
Family Assessment Device (total score), mean (SD)	1.9 (0.4)	2.0 (0.4)	0.12

ERC, Emotion Regulation Checklist; WCST, Wisconsin Card Sorting Test. *p*-values are derived from independent samples *t*-tests for continuous data and chi-square tests for categorical data. No significant differences were found at baseline (*p* > 0.05), indicating successful randomization.

### 3.2 Effects of mindfulness education on children’s emotion regulation

Results from the repeated-measures ANCOVA, summarized in Table 2, showed that mindfulness education significantly improved children’s emotion regulation capacity. Regarding the primary outcome measure, the repeated-measures ANCOVA revealed a significant time × group interaction (*F* = 15.37, *p* < 0.001, partial *η*^2^ = 0.101), indicating that the children in the mindfulness group demonstrated much greater improvement in emotion regulation strategy scores than those in the control group. The difference between the two groups was significant at post-test (*p* = 0.003).

**Table 2:** Effects of mindfulness education on children’s emotion regulation outcomes.

Outcome measure	Mindfulness group (*n* = 70)	Control group (*n* = 70)	Group effect	Time × group Interaction					
	Baseline	Post-test	Baseline	Post-test	*F* value	p-value	*F* value	p-value	Partial *η*^2^
Primary outcome									
ERC Score	78.5 (12.3)	85.2 (10.1)	76.8 (11.9)	77.1 (12.5)	8.92	0.003	15.37	< 0.001	0.101
Secondary outcomes									
Emotion Management Scale	65.2 (15.1)	72.8 (12.3)	67.1 (14.5)	68.3 (13.8)	5.24	0.024	9.85	0.002	0.067
Disappointing Gift Task	3.8 (1.2)	2.9 (1.0)	3.9 (1.1)	3.7 (1.2)	6.73	0.01	12.41	0.001	0.083

ERC, Emotion Regulation Checklist. All values are presented as mean (SD). Analysis was conducted using a repeated-measures ANCOVA, controlling for baseline scores. Partial *η*^2^ represents effect size (0.01 = small, 0.06 = medium, 0.14 = large).

**Figure 2:**
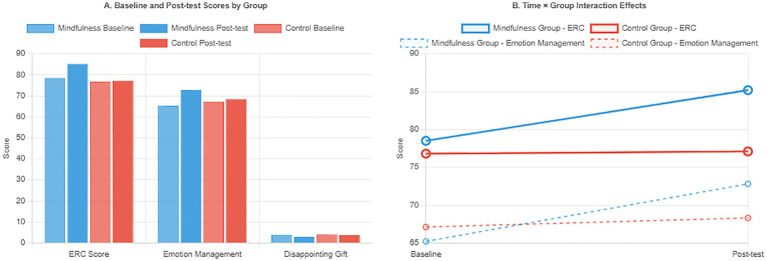
Effects of mindfulness education on children’s emotion regulation outcomes.

About the secondary measures, the results also demonstrated much greater improvement in the two emotional behavioral responses: the teacher-rated Emotion Management Scale exhibited a significant interaction effect (*F* = 9.85, *p* = 0.002, partial *η*^2^ = 0.067), and the negative expression scores in the Disappointing Gift Task also demonstrated a significant interaction (*F* = 12.41, *p* = 0.001, partial *η*^2^ = 0.083). All effect sizes were medium, indicating that mindfulness education significantly improves children’s emotion regulation capacity (Figure 2).

### 3.3 Effects of mindfulness education on children’s executive function

**Figure 3:**
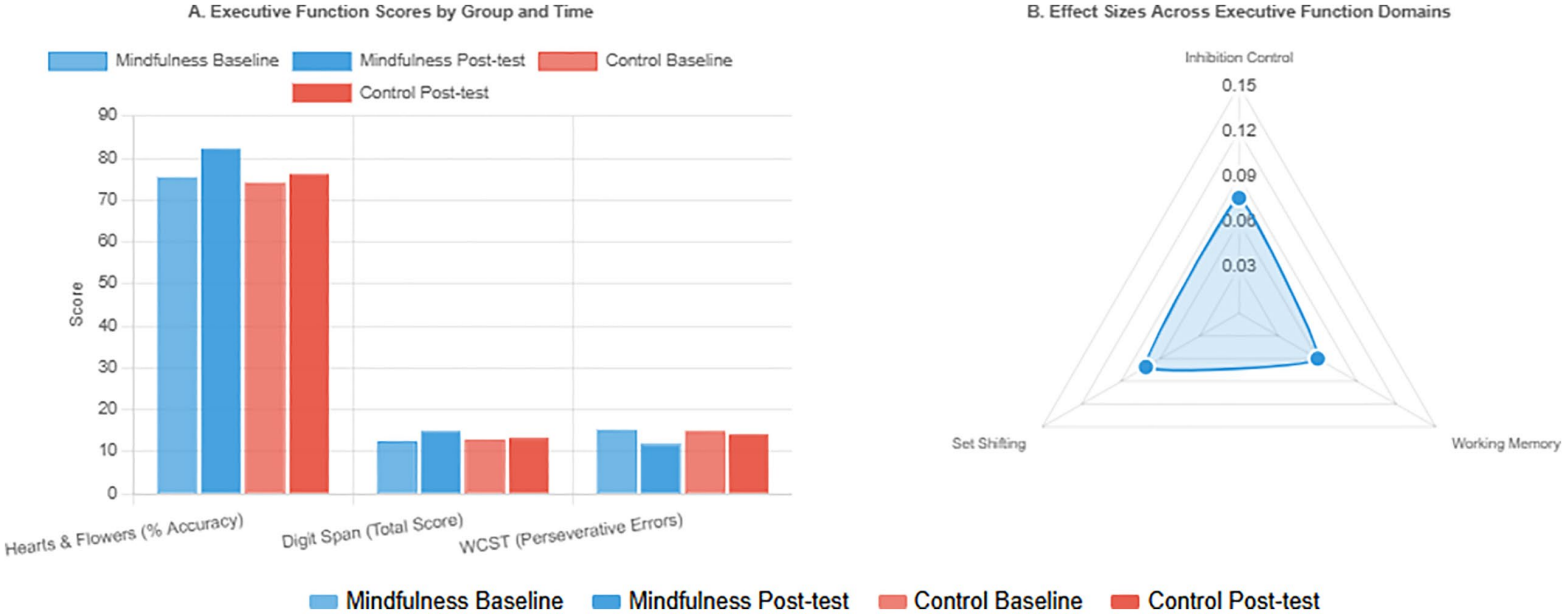
Effects of mindfulness education on children’s executive function outcomes.

As shown in Table 3, a repeated-measures ANCOVA revealed that mindfulness education produced significant improvements across all components of children’s executive function. For inhibition control and cognitive flexibility, a significant time × group interaction was found in the accuracy rate of the Hearts and Flowers task (*F* = 11.29, *p* = 0.001, partial *η*^2^ = 0.076), with the mindfulness group demonstrating substantially greater improvement compared to the control group, and the between-group difference reaching statistical significance (*p* = 0.008). Regarding working memory, the Digit Span Task scores also showed a significant interaction effect (*F* = 8.74, *p* = 0.004, partial *η*^2^ = 0.060), indicating more pronounced improvement in working memory capacity in the mindfulness group. For set-shifting ability, perseverative errors on the Wisconsin Card Sorting Test showed a significant interaction (*F* = 10.56, *p* = 0.001, partial *η*^2^ = 0.071), with the mindfulness group showing a significantly greater reduction in errors than the control group. All effect sizes were medium, confirming that mindfulness education effectively improves children’s core executive functions (Figure 3).

**Table 3:** Effects of mindfulness education on children’s executive function outcomes.

Executive function measure	Mindfulness group (*n* = 70)	Control group (*n* = 70)	Group effect	Time × group interaction					
	Baseline	Post-test	Baseline	Post-test	*F* value	*p*-value	*F* value	*p*-value	Partial *η*^2^
Inhibition control and cognitive flexibility									
Hearts and Flowers (% accuracy)	75.4 (10.2)	82.3 (8.7)	74.1 (9.8)	76.2 (9.5)	7.15	0.008	11.29	0.001	0.076
Working memory									
Digit Span Task (total score)	12.5 (3.1)	14.8 (2.8)	12.8 (2.9)	13.2 (3.0)	5.89	0.016	8.74	0.004	0.06
Set shifting									
WCST (perseverative errors)	15.2 (4.5)	11.8 (3.9)	14.8 (4.9)	14.1 (4.3)	6.42	0.012	10.56	0.001	0.071

WCST, Wisconsin Card Sorting Test. All values are presented as mean (SD). Analysis was conducted using a repeated-measures ANCOVA, controlling for baseline scores. Partial *η*^2^ represents effect size (0.01 = small, 0.06 = medium, 0.14 = large).

### 3.4 Mediation analysis of executive function in the pathway from intervention to emotion regulation

The mediation analysis output is shown in Table 4. Executive function behavior served as an important mediator of the effect of the mindfulness intervention on children’s emotion regulation. The total indirect effect was 3.67 (Bootstrapped 95% CI: LLCI = 2.42, ULCI = 5.08, *p* < 0.001), which explained 53.8% of the total effect. The mediating effect of inhibitory control (effect = 1.28, *p* = 0.002), working memory (effect = 0.89, *p* = 0.008), and cognitive flexibility (effect = 1.50, *p* = 0.001) was all statistically significant, and the mediating effect of cognitive flexibility was the strongest. These results suggested that mindfulness education affected children’s emotion regulation not only through direct effects but also through indirect effects via improvements in the three executive function components, further validating the multiple mediation effect of executive function in the relationship between mindfulness and emotion regulation.

**Table 4:** Mediation analysis of executive function in the relationship between mindfulness intervention and emotion regulation improvement.

Mediation pathway	Effect type	Estimate	SE	Boot LLCI	Boot ULCI	*p*-value
Total effect	Mindfulness → ERC Score	6.82	1.23	4.39	9.25	<0.001
Direct effect	Mindfulness → ERC Score	3.15	1.05	1.08	5.22	0.003
Indirect effects						
Through inhibition control	Mindfulness → Hearts and Flowers → ERC	1.28	0.41	0.58	2.15	0.002
Through working memory	Mindfulness → Digit Span → ERC	0.89	0.32	0.33	1.58	0.008
Through set shifting	Mindfulness → WCST → ERC	1.5	0.45	0.72	2.45	0.001
Total indirect effect	Combined mediation	3.67	0.68	2.42	5.08	<0.001

ERC, Emotion Regulation Checklist; WCST, Wisconsin Card Sorting Test; SE, standard error; Boot LLCI/ULCI, bias-corrected bootstrap 95% confidence interval (5,000 samples). All pathways control for baseline scores, age, gender, parental education, and family functioning. The mediation model explains 22.5% of the variance in emotion regulation improvement (*R*^2^ = 0.225).

## 4 Discussion

### 4.1 Mindfulness education effectively enhances children’s emotion regulation and executive function

Results from this study showed that an 8-week mindfulness education program enhanced emotion regulation and executive function in school-aged children, supporting the primary hypothesis. Regarding emotion regulation, compared with those in the control group, children in the mindfulness group showed greater gains in the use of emotion regulation strategies and teacher-rated externalizing behaviors at school, and exhibited different emotional-behavioral responses during the laboratory assessment. These results are consistent with previous studies, confirming that mindfulness training helps children be more nonjudgmentally aware of their present-moment experiences, acknowledge the fluctuation of emotions without being impelled by them in the moment, and hence achieve more efficient cognitive and behavioral emotion management (Spencer et al., 2023; Pickerell et al., 2023; Rowland et al., 2023; Zenner et al., 2014; Phan et al., 2022; Shapiro et al., 2006).

More importantly, this is the first study, to our knowledge, to demonstrate the synergistic enhancement of the three basic components of executive function, inhibitory control, working memory, and cognitive flexibility, in a single framework, through mindfulness education. Mindfulness training that asks children to attend to the breath trains inhibitory control; gently redirecting attention when the mind wanders strengthens working memory and cognitive flexibility; and children adopting an open attitude toward the shift in internal experiences in the moment cultivates cognitive flexibility (Chen et al., 2022; Zebdi et al., 2021). In other words, the above three basic components of executive function repeatedly activate and strengthen neural circuits. Subjective rating scales, in addition to objective behavioral tasks, are used in this study, which provides strong empirical support for the claim that mindfulness training is a practical approach to enhancing executive function in children. In contrast, previous research has focused mainly on adults or on a single aspect of executive function.

### 4.2 Executive function as a core mechanism linking mindfulness and emotion regulation

The mediation analysis demonstrates that executive function serves as a significant mediator in the relationship between mindfulness education and children’s emotion regulation. The mediation analysis demonstrates that executive function serves as a significant mediator in the relationship between mindfulness education and children’s emotion regulation (Groves et al., 2021; Pozo-Rodríguez et al., 2026)^.^ Rather than operating solely through direct pathways, mindfulness training substantially enhances emotion regulation by improving three core executive function components: inhibitory control, working memory, and cognitive flexibility. The identified “mindfulness → executive function → emotion regulation” pathway reveals specific cognitive mechanisms through which mindfulness exerts its effects.

Enhanced inhibitory control enables children to pause when emotional impulses arise, creating crucial temporal and psychological space for subsequent cognitive regulation (Deplus et al., 2016; Ren et al., 2020; Department of Psychology, Shahid Bahonar University, Kerman, Iran, 2020). Improved working memory allows children to simultaneously maintain emotional experiences and long-term goals, such as maintaining social harmony, thereby facilitating more adaptive regulatory strategies (Gouveia et al., 2019; Children and Adolescent Mental Health Research Group, Institut de Recerca Sant Joan de Déu, Barcelona, Spain, 2019). Increased cognitive flexibility helps children disengage from negative emotional contexts and flexibly shift perspectives, reducing rigid, maladaptive response patterns.

This study advances self-regulation research by connecting macro-level behavioral outcomes with underlying cognitive mechanisms—executive function—and linking these mechanisms to an evidence-based educational intervention. The complete pathway from intervention to mechanism to outcome provides a new theoretical framework for understanding and promoting children’s psychological health.

### 4.3 Cultural considerations and cross-cultural components of mindfulness

Although culture can shape how mindfulness is perceived and accepted, core components of mindfulness are generally regarded as cross-culturally applicable, including present-moment attention regulation and a nonjudgmental, accepting stance toward internal experiences (Shapiro et al., 2006). These core skills are not dependent on culture-specific beliefs and can be delivered as practical self-regulation strategies in structured school contexts (Zenner et al., 2014; Phan et al., 2022). In the present setting, the brief, skills-focused classroom format and its alignment with daily school routines may help explain the program’s feasibility and its benefits for children’s executive function and emotion regulation. This framing clarifies why mindfulness-based education can be successful in this context despite potential cultural differences in attitudes toward mindfulness.

### 4.4 Limitations and future directions

Despite the rigorous design, several limitations warrant consideration. First, the sample from specific regional community schools limits generalizability, necessitating future validation across broader geographical, cultural, and clinical populations, including children with anxiety or ADHD. Second, while incorporating objective behavioral tasks, the study relied primarily on parent and teacher reports for emotion regulation assessment. Future research would benefit from incorporating physiological measures such as heart rate variability and EEG to establish a more comprehensive assessment framework. Third, the exclusive focus on short-term post-intervention effects leaves unanswered questions about the long-term sustainability of mindfulness benefits and associated neural changes.

Future research should prioritize three key directions. Longitudinal follow-up studies are essential to examine the sustained durability of mindfulness effects. Neuroimaging approaches, exceptionally functional magnetic resonance imaging (fMRI), could elucidate the neural substrates through which mindfulness influences brain development in children. From an applied perspective, research should explore practical strategies for integrating mindfulness education into standard school curricula and investigate personalized intervention approaches tailored to individual characteristics, such as temperament and baseline executive function levels.

## 5 Conclusion

This randomized controlled trial demonstrates that a structured mindfulness education program effectively enhances emotion regulation and executive function in school-aged children. Importantly, mediation analyses revealed that executive function serves as a central mechanism linking mindfulness training to improvements in emotion regulation, operating through three parallel pathways: inhibitory control, working memory, and cognitive flexibility. These findings elucidate the underlying cognitive-neural pathway of mindfulness efficacy, showing that systematic training and reinforcement of core cognitive functions facilitate the development of children’s emotion self-regulation. The results provide empirical support for the theoretical pathway of “mindfulness → executive function → emotion regulation” and offer a scientific basis for the implementation of mindfulness education in school settings to promote children’s psychological health and socio-emotional development.

## Data Availability

The raw data supporting the conclusions of this article will be made available by the authors, without undue reservation.
